# The Association between Newborn Regional Body Composition and Cord Blood Concentrations of C-Peptide and Insulin-Like Growth Factor I

**DOI:** 10.1371/journal.pone.0121350

**Published:** 2015-07-07

**Authors:** Emma M. Carlsen, Kristina M. Renault, Rikke B. Jensen, Kirsten Nørgaard, Jens-Erik B. Jensen, Lisbeth Nilas, Dina Cortes, Kim F. Michaelsen, Ole Pryds

**Affiliations:** 1 Department of Pediatrics, Hvidovre University Hospital, University of Copenhagen, Hvidovre, Denmark; 2 Department of Obstetrics and Gynecology, Odense University Hospital, University of Southern Denmark, Odense, Denmark; 3 Department of Growth and Reproduction, Rigshospitalet, Faculty of Health and Medical Sciences, University of Copenhagen, Copenhagen, Denmark; 4 Department of Endocrinology, Hvidovre University Hospital, University of Copenhagen, Hvidovre, Denmark; 5 Faculty of Health Science, University of Copenhagen, Copenhagen, Denmark; 6 Department of Obstetrics and Gynecology, Hvidovre University Hospital, University of Copenhagen, Hvidovre, Denmark; 7 Department of Nutrition, Exercise and Sports, Faculty of Science, University of Copenhagen, Copenhagen, Denmark; Pennington Biomed Research Center, UNITED STATES

## Abstract

**Background:**

Third trimester fetal growth is partially regulated by C-peptide and insulin-like growth factor I (IGF-I). Prenatal exposures including maternal obesity and high gestational weight gain as well as high birth weight have been linked to subsequent metabolic disease. We evaluated the associations between newborn regional body composition and cord blood levels of C-peptide and IGF-I.

**Methods:**

We prospectively included obese and normal-weight mothers and their newborns; cord blood was collected and frozen. Analyses of C-peptide and IGF-I were performed simultaneously, after recruitment was completed. Newborn regional body composition was assessed with dual-energy X-ray absorptiometry scanning (DXA) within 48 hours of birth.

**Results:**

Three hundred thirty-six term infants were eligible to participate in the study; of whom 174 (52%) infants had cord blood taken. Total, abdominal and arm and leg fat mass were positively associated with C-peptide (*p* < 0.001). Arm and leg fat mass was associated with IGF-I concentration: 28 g [95% confidence interval: 4, 53] per doubling of IGF-I. There was no association between total or abdominal fat mass and IGF-I. Fat-free mass was positively associated with both C-peptide (*p* < 0.001) and IGF-I (*p* = 0.004).

**Conclusion:**

Peripheral fat tissue accumulation was associated with cord blood C-peptide and IGF-I. Total and abdominal fat masses were related to C-peptide but not to IGF-I. Thus, newborn adiposity is partially mediated through C-peptide and early linear growth is associated with IGF-I.

## Introduction

The prevalence of childhood obesity has increased dramatically during the last decades in high as well as low income settings [1]. The prenatal and early postnatal periods have been identified as sensitive periods during which the susceptibility of developing obesity is affected by genetic, epigenetic, and environmental factors [2–5].

The offspring of obese mothers have an increased risk of becoming obese compared to offspring of normal-weight mothers [6]. A less favorable intrauterine environment may play an important role in development of obesity [7,8]. Increased pre-pregnancy body mass index (BMI), excessive gestational weight gain (GWG), and elevated glucose levels are known to independently influence fetal growth and tissue composition, and to increase the risk of subsequent development of obesity [9–13]. Thus maternal phenotype and life style may contribute to a vicious trans-generational cycle in which the risk of obesity is acquired through both genetics and exposures.

Population-based preventive strategies against childhood obesity seem less promising [14], and therefore detailed knowledge about risk factors is imperative in order to design future interventional studies targeting childhood obesity [4].

Insulin and insulin-like growth factor I (IGF-I) are the most important growth factors in the regulation of second and third trimester fetal growth [15]. C-peptide may be used as a proxy for insulin in cohort studies since it is more stable than insulin [16]. Fetal C-peptide is related to maternal glycemic control during pregnancy [17], a theory originally proposed by Pedersen et al. in 1952. Glucose transverses the placenta and promotes insulin (and C-peptide) secretion in the fetus [18,19]. Female sex, high birth weight, and adiposity have been linked to an elevated concentration of C-peptide in newborns [17]. C-peptide stimulates the release of IGF-I in the fetus [20]. IGF-I is a polypeptide that affects cellular proliferation and differentiation and has in previous studies been positively associated with female sex, birth weight, and birth length [15,21,22]. IGF-I may influence fat cell differentiation, an in vitro study found that the differentiation of visceral fat cells is strongly associated with IGF-I than with subcutaneous fat cells [23]. Whereas fetal C-peptide is modifiable through maternal glucose levels, IGF-I is more constant and to a wider extent genetically determined [24].

Fetal levels of anabolic hormones may be the common pathway between the intrauterine environment and body composition in the newborn and subsequent risk of metabolic disease [6,9,25]. This is the first study to evaluate the association between newborn regional body composition and cord blood levels of C-peptide and IGF-I. We hypothesize that the amount of abdominal fat mass is primarily associated with IGF-I and that the other fat mass depots (arm and leg fat mass) are closer associated with C-peptide

## Methods

### Study design

Obese (n = 130) and normal-weight (n = 44) mothers and their offspring were included in the study. Obese mothers were recruited at the time of the first ultrasound examination in gestational week 11–13 in the Treatment of Obese Pregnant (TOP) study at Hvidovre Hospital, Copenhagen University (reported in detail elsewhere [26]). The TOP study inclusion criterion was a pre-pregnancy BMI above or equal to 30 kg/m^2^, and the obese pregnant women were encouraged to keep GWG below 5 kg. Obese mothers were randomized to: PA+D: physical activity intervention assessed by pedometer and diet, PA: physical activity intervention assessed by pedometer or C: control (the control group was also advised to limit GWG according to the TOP-study recommendation). The TOP study included a total of 425 women. Recruitment for this study was initiated 1 year and 7 months after the TOP study started at which time 135 women had already given birth, leaving 284eligible for inclusion in the present study.

The group of normal weight mothers was selected on 15 random days from the group of pregnant women visiting the maternity ward for routine check-ups in the third trimester and who were not in labor. Normal weight was defined as a self-reported pre-pregnancy BMI between 18.5–24.9 kg/m^2^.

We included healthy, newborn, singleton infants born at term (>258 days of gestation), and with a postnatal age < 48 hours. Infants were recruited between December 10, 2010 and June 30, 2012. We excluded mothers with a chronic disease and pre-eclampsia. In Denmark, gestational diabetes mellitus (GDM) is defined as 2-h glucose > 8.9 mmol/l [27]. Women diagnosed with GDM were excluded. Infants requiring admission to a neonatal intensive care unit or suffering from congenital diseases or malformations were excluded.

#### Maternal data

Information on maternal educational and smoking status, exercise habits, and any previous pregnancies was collected from a questionnaire filled in during the first trimester survey for obese mothers and during the third trimester for normal-weight mothers. Gestational age was determined through an early ultrasound examination (gestational week 11–13).

Pre-pregnancy weight was self-reported. All women were weighed at the hospital wearing light clothing and no shoes at 36–37 week of gestation (Seca digital scales, Germany). GWG was calculated as the difference between pre-pregnancy weight and weight at 36–37 weeks of gestation. GWG was categorized based on Institute of Medicine’s (IOM) criteria into inadequate, adequate, or excessive weight gain. Adequate GWG is 5–9 kg for pre-pregnancy obese and 11.5–15.9 for pre-pregnancy normal weight [28]. Mode of delivery was categorized as vaginal delivery, assisted vaginal delivery, acute caesarean section and elective caesarean section.

#### Infant data

The newborns were weighed recumbent on an infant scale (Seca 727, digital baby scales Germany). Length and head circumference were measured with non-stretchable measuring tape according to WHO guidelines [29]. Abdominal circumference was measured by non-stretchable measuring tape during mid-expiration at the umbilical level in the supine position.

#### Body composition assessment

Infant body composition was assessed using dual-energy X-ray absorptiometry (DXA) scanning (DXA, Hologic 4500, Bedford, MA, USA) within 48 hours after birth. Through this method information on fat and fat-free body mass as well as bone mass was obtained. When evaluating the scans, we used the same criteria as Cooke et al. [30], and only scans that met predefined quality criteria were included in the analysis. Fat mass/total mass was used to calculate fat%.

Abdominal fat mass and fat-free mass were estimated by identifying two abdominal regions using Pediatric DXA software from Hologic. The first region was limited by the thoracic diaphragm and both upper iliac crests, the second region was limited laterally by the upper iliac crests and caudally by the femoral heads, and the total sum of the two regions constituted the abdominal region. Arm and leg fat mass were calculated by defining all four extremities as regions and thereafter calculating a total mass. From duplicate scans in 58 infants, the test-retest variability concerning DXA-derived fat and fat-free mass was calculated to 11.8% and 7.1%, respectively (data not shown).

Birth weight z-score is birth weight adjusted for gestational age and sex and was calculated using Marsal et al. as normal reference [31].

#### Cord blood samples

Cord blood samples were obtained immediately after birth and after clamping the umbilical vein, only vein blood was obtained. The blood was not milked. Samples were obtained by the staff at the maternity ward. Samples were sent for immediate centrifugation, and the plasma was separated and stored at -80°C until analyses were performed. C-peptide and IGF-I were analyzed as a batch using an Immulite 1000 Analyzer (Diagnostic Products Corporation, Los Angeles, CA, USA) with kits from Siemens, (Siemens healthcare, Germany). The detection limit for IGF-I was 25 ng/mL. Values below the detection limit were coded 24 ng/mL (to diminish variance); this was the case in 14 samples. If extensive hemolysis was present, C-peptide was not analyzed due to the uncertainty this imposes (seven samples were excluded due to hemolysis).

#### Ethics

Informed written consent was obtained from both parents before infants were included in the study, and the Ethics Committee of the Capital Region of Denmark (H-D-2008-119) approved the study. The study was registered at www.clinicaltrials.gov (NCT01235676) and performed according to the Helsinki II declaration.

#### Statistical analysis

Overall baseline characteristics for pre-pregnancy obese mothers and normal-weight mothers and their offspring are presented as mean with standard deviation (SD), median with range, or proportions, (Table 1). Differences in cord blood hormones between sexes and adjusted for gestational age were calculated by multiple regression. C-peptide and IGF-I were transformed using the natural logarithm to obtain homogeneity of variances. The correlation between C-peptide and IGF-I was assessed using Spearman’s correlation. The influence of TOP-study randomization on C-peptide and IGF-I and newborn anthropometrics were assessed using multiple regression analyses, the models were adjusted for maternal age, pre-pregnancy BMI, GWG, parity, smoking, education, mode of delivery, gestational diabetes mellitus, newborn gestational age, and sex.

**Table 1:** Baseline characteristics, maternal and newborn, grouped according to maternal pre-pregnant body mass index (BMI) (kg/m^2^).

Maternal characteristics	Obese mothers	Normal weight mothers	*p* value
	*n* = 130	*n* = 44	
Maternal age (years) ^1^	31.0 (4.4)	31.2 (4.6)	0.75
Pre-pregnant BMI (kg/m^2^) ^2^	34.4 (30.0–50.3)	22.2 (19.0–24.9)	< 0.001
Gestational weight gain (kg) ^1^	10.1 (6.3)	14.1 (3.6)	< 0.001
IOM GWG categories (n) ^3^			
Inadequate	29	11	
Adequate	33	24	
Excessive	68	9	< 0.001
Primiparas (%)^3^	61	66	0.92
Maternal education (years) ^1^	14.5 (2.3)	15.1 (2.3)	0.14
Maternal smoking (%)^3^	10	7	0.53
Mode of delivery			
Vaginal	76	26	
Assisted Vagnial	13	4	
Acute ceaseran section	26	4	
Elective ceaseran section	15	10	0.16
Newborn characteristics			
Birth weight (g) ^1^	3728 (525)	3572 (462)	0.082
Birth length (cm) ^1^	52.7 (2.3)	51.9 (2.0)	0.043
Head circumference (cm) ^1^	35.4 (1.6)	34.8 (1.7)	0.037
Abdominal circumference (cm) ^1^	33.7 (2.1)	33.4 (1.8)	0.33
Sex ^3^			
Male (%)	55	45	
Female (%)	45	55	0.29
Gestational age at birth (days) ^1^	281 (9)	281 (8)	0.74
Birth weight z-score * ^1^	0.30 (1.20)	-0.05 (0.86)	0.041
Fat-free mass (g) ^1^	3404 (399)	3390 (393)	0.85
Fat mass (g) ^1^	436 (221)	342 (163)	0.01
Fat (%)^1^	11.0 (4.4)	8.9 (3.5)	0.004
Abdominal fat mass (g) ^1^	57 (31)	42 (22)	0.001
Arm and leg fat mass (g) ^1^	232 (113)	176 (76)	<0.001
IGF-I (ng/mL) ^1^	71.5 (31.8)	67.0 (24.7)	0.35
Log IGF-I^1^	4.2 (0.5)	4.1 (0.4)	0.70
C-peptide (pmol/l)^2^	442 (76–1427)	396 (102–1526)	0.13
Log C-peptid^1^	5.9 (0.6)	5.8 (0.6)	0.19
Apgar (5 min)^2^	10 (7–10)	10 (8–10)	0.35
Birth weight > 4000 g^3^% (n)	23 (10)	29 (38)	0.40
Birth weight < 2500 g^3^% (n)	0 (0)	2 (3)	0.30

^1^ Mean (±SD), Student’s *t*-test

^2^ Median (range), Mann-Whitney test

^3^Proportion, chi-square test

*Birth weight adjusted for gestational age at birth and sex, according to Marsal et al.

Multiple linear regressions was used to evaluate the effect of C-peptide and IGF-I on birth weight z-score, newborn body composition, newborn fat mass, fat-free mass, fat%, abdominal fat mass, and arm and leg fat mass. All models are presented as raw values adjusted for newborn sex and gestational age or fully adjusted for maternal age, pre-pregnancy BMI, GWG, parity, smoking, education, mode of delivery, newborn gestational age, and sex.

To determine whether maternal factors (pre-pregnancy obesity, GWG, and parity) influenced cord blood C-peptide and IGF-I concentrations, multiple linear regression analysis was performed, fully adjusted for maternal age, smoking, education, mode of delivery, newborn gestational age, and sex. IGF-I concentration was also adjusted for C-peptide levels. The level of significance was set to 0.05 (SPSS Statistics, version 19.0, Chicago, IL, USA).

## Results

**Fig 1:**
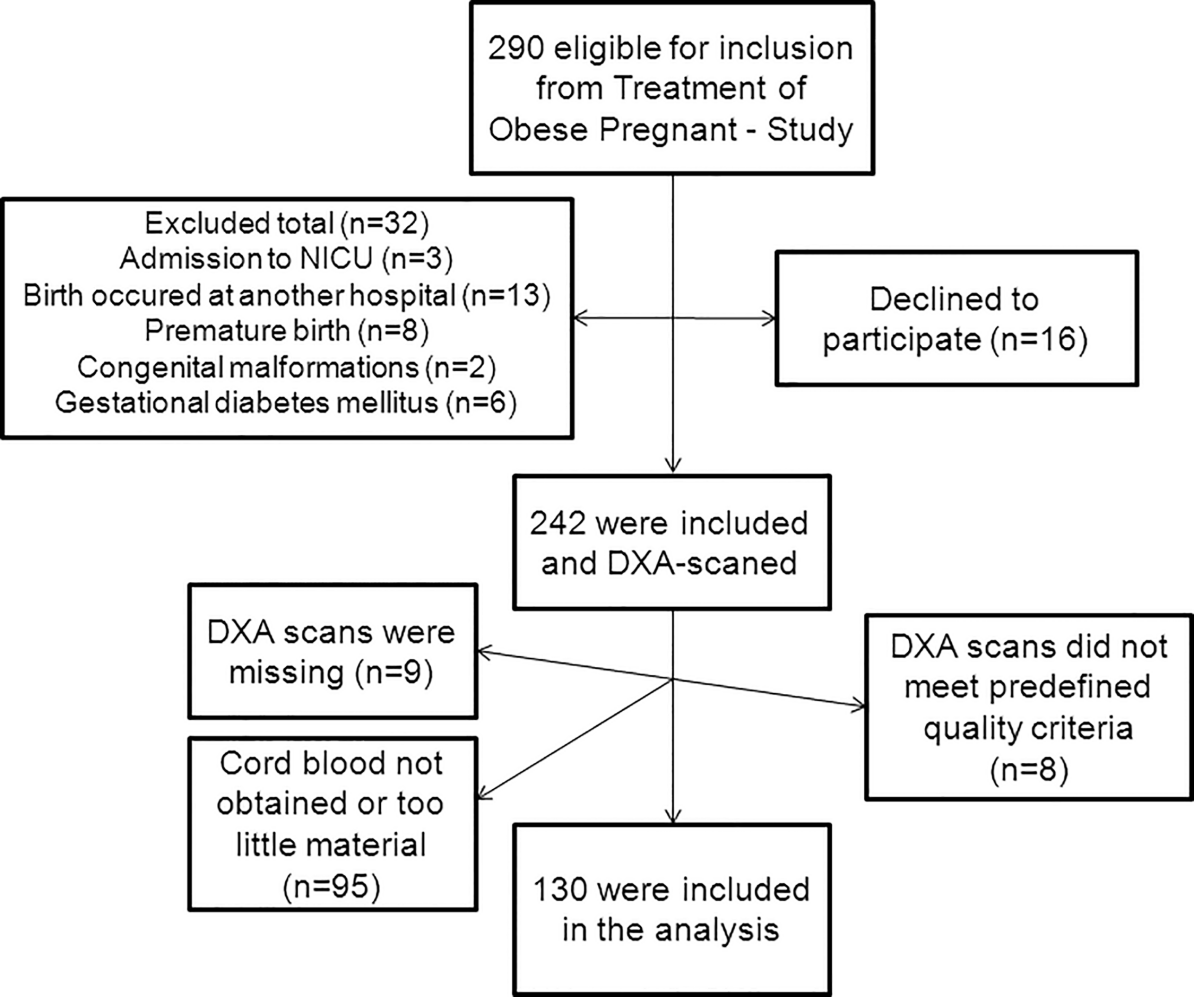
Study inclusion pre-pregnancy obese women. When too little cord blood was obtained, it was not possible to analyze C-peptide and IGF-I.

**Fig 2:**
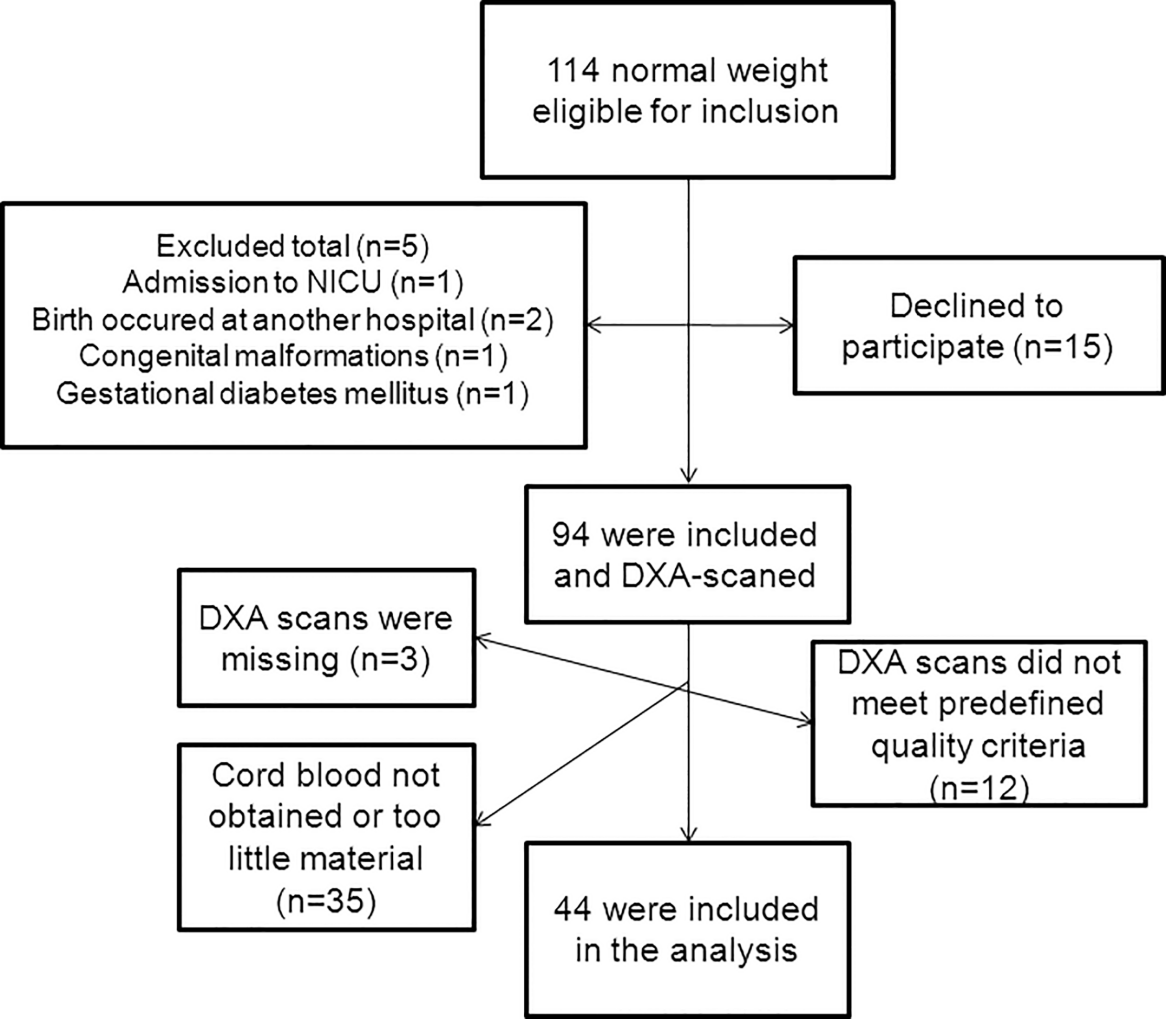
Study inclusion pre-pregnancy normal weight women. When too little cord blood was obtained, it was not possible to analyze C-peptide and IGF-I.

In total 336 were eligible for inclusion and of these 174 (52%) were included and had cord blood samples taken (Figs 1 and 2).

There were no differences in pre-pregnancy BMI, GWG, and fetal birth weight between the participants who had cord blood taken compared to those who did not. Concentrations of C-peptide were positively correlated to the concentrations of IGF-I (*r* = 0.15, *p* = 0.044).

The TOP-study randomization did not affect cord blood concentration of C-peptide and IGF-I (data not shown) nor did it affect newborn anthropometric measurements [32].

### Clinical characteristics of obese versus pre-pregnancy normal-weight mothers and their offspring

A larger proportion of pre-pregnancy obese mothers had an excessive GWG as defined by IOM [28], but mean GWG was lower in the obese than in the normal weight mothers (*p* < 0.001), (Table 1). Except for the pre-pregnancy BMI and GWG, there were no other differences in baseline characteristics between obese and normal-weight mothers.

The infants of the obese mothers had a higher mean birth weight z-score of 0.35, (*p* = 0.041) and a greater mean fat mass of 94 g (*p* = 0.001) than offspring of normal-weight mothers. There was no difference in newborn fat-free mass (*p* = 0.85) (Table 1). IGF-I concentrations were higher in female infants (*p* = 0.014), whereas there was no differences in C-peptide concentrations between the sexes. (Table 2).

**Table 2:** Cord blood concentration of c-peptide and IGF-I, presented stratified by sex.

	Boys (*n* = 91)	Girls (*n* = 83)	*p* value
C-peptide (pmol/l)^1^	374 (76–1427)	387 (97–1526)	0.38
Log C-peptide^2^	5.9 (0.5)	6.0 (0.6)	0.31
IGF-I (ng/mL) ^2^	65.0 (26.4)	76.2 (33.0)	0.014
Log IGF-I^2^	4.1 (0.4)	4.2 (0.5)	0.037

^1^ Median (range)

^2^ Mean (±SD), Student’s *t*-test

### Newborn regional body composition versus C-peptide and IGF-I

Birth weight z-score and birth length were positively associated with both C-peptide and IGF-I. Total fat mass, abdominal fat mass, and arm and leg fat mass were significantly related to C-peptide, both unadjusted and adjusted. Per doubling of the concentration of C-peptide, fat % increased by 1.3%, [95% confidence interval (CI): 0.6, 2.0], (*p* < 0.001). In contrast, only arm and leg fat mass but not the abdominal fat mass was related to IGF-I concentration. Per doubling of IGF-I concentration, the adjusted arm and leg fat mass increased by 28g [95% CI: 4, 53] (*p* = 0.023), but fat % was not associated with cord blood IGF-I levels.

Newborn fat-free mass was related to the concentration of C-peptide and IGF-I; per doubling of C-peptide fat-free mass increased with 157 g [95% CI: 95, 219] and per doubling of the concentration of IGF-I, the fat-free mass increased with 121 g [95% CI: 39, 204]. These analyses were adjusted for maternal age, education, smoking, parity, pre-pregnancy BMI, GWG, mode of delivery and newborn gestational age and sex (Tables 3, 4, 5, and 6).

**Table 3:** The association between birth weight (z-score) and newborn fat (%) and C-peptide and IGF-I.

	Dependent							
	Birth weight (z-score)		Birth weight (z-score)		Fat (%)		Fat (%)	
	(Unadjusted)		(Fully adjusted*)		(Partly adjusted**)		(Fully adjusted***)	
	ß [95% CI]	*p* value	ß [95% CI]	*p* value	ß [95% CI]	p- value	ß [95% CI]	p-value
Independents								
Constant	-8.0		-12.2		-34		-51	
C-peptide^1^	0.8 [0.6, 1.1]	**<0.001**	0.6 [0.4, 0.8]	**<0.001**	2.4 [1.3, 3.4]	**<0.001**	1.9 [0.8, 2.9]	<**0.001**
IGF-I^1^	0.8 [0.5, 1.1]	**<0.001**	0.4 [0.1, 1.6]	**0.005**	0.8 [-0.6, 2.1]	0.26	0.9 [-0.5, 2.2]	0.20

^1^ Log-values

*Adjusted for maternal age, education, smoking, parity, pre-pregnancy BMI, GWG, mode of delivery and birth length

**Adjusted for gestational age and sex

***Adjusted for maternal age, education, smoking, parity, pre-pregnancy BMI, GWG, mode of delivery and newborn gestational age and sex

**Table 4:** The association between newborn total fat-free mass and fat mass and C-peptide and IGF-I.

	Dependent							
	Fat-free mass (g)		Fat-free mass (g)		Fat mass (g)		Fat mass(g)	
	(Partly adjusted*)		(Fully adjusted**)		(Partly adjusted*)		(Fully adjusted**)	
	ß [95% CI]	*p* value	ß [95% CI]	*p* value	ß [95% CI]	p- value	ß** [95% CI]	p-value
Independents								
Constant	-2963		-3058		-2266		-3030	
C-peptide^1^	226 [137, 315]	**<0.001**	227 [137, 317]	**<0.001**	135 [84, 185]	**<0.001**	111 [61, 161]	**<0.001**
IGF-I^1^	236 [124, 349]	**<0.001**	176 [56, 295]	**0.004**	48 [-16, 112]	0.14	45 [-21, 111]	0.18

^1^ Log-values

*Adjusted for gestational age and sex

**Adjusted for maternal age, education, smoking, parity, pre-pregnancy BMI, GWG, mode of delivery and newborn gestational age and sex

**Table 5:** The association between arm and leg fat mass and abdominal fat mass and C-peptide and IGF-I.

	Dependent							
	Abdominal Fat mass (g)		Abdominal Fat mass (g)		Arm and Leg Fat Mass (g)		Arm and Leg Fat Mass (g)	
	(Partly adjusted*)		(Fully adjusted**)		(Partly adjusted*)		(Fully adjusted**)	
	ß [95% CI]	*p* value	ß [95% CI]	*p* value	ß [95% CI]	p- value	ß [95% CI]	p-value
Independents								
Constant	-263		-364		-1094		-1197	
C-peptide^1^	16 [9,24]	**<0.001**	13 [6,21]	**0.001**	68 [40, 92]	**<0.001**	57 [31, 83]	**<0.001**
IGF-I^1^	2 [–8,11]	0.75	0.4 [–9,10]	0.93	48 [13, 82]	**0.007**	41 [6, 77]	**0.023**

^1^ Log-values

*Adjusted for gestational age and sex

**Adjusted for maternal age, education, smoking, parity, pre-pregnancy BMI, GWG, mode of delivery and newborn gestational age and sex

**Table 6:** The association between birth length and C-peptide and IGF-I.

	Dependent			
	Birth length (cm)		Birth length (cm)	
	(Partly adjusted*)		(Fully adjusted**)	
	ß [95% CI]	*p* value	ß [95% CI]	*p* value
Independents				
Constant	23.27		20.6	
C-peptide^1^	0.89 [0.34, 1.45]	**0.002**	0.83 [0.27, 1.40]	**0.004**
IGF-I^1^	0.90 [0.20, 1.60]	**0.012**	0.81 [0.06, 1.56]	**0.034**

^1^ Log-values

*Adjusted for gestational age and sex

**Adjusted for maternal age, education, smoking, parity, pre-pregnancy BMI, GWG, mode of delivery and newborn gestational age and se

### C-peptide and IGF-I versus maternal factors

The concentration of C-peptide was positively associated with GWG and pre-pregnancy obesity. Per kg increase in GWG, C-peptide rose by a mean of 2.2% [95% CI: 0.7, 3.7]. In obese women, C-peptide increased by 22.1% [95% CI: 1.7, 42.5]. IGF-I was not associated with GWG or obesity. IGF-I were inversely associated with parity and levels were 29.1% lower in primiparas [95% CI: -43.0, -15.0] (Table 7).

**Table 7:** The association between cord blood concentrations of C-peptide and IGF-I and maternal pre-pregnancy obesity, GWG, and primiparity.

	Dependents			
	C-peptide^1^ *		IGF-I^1^ **	
	ß [95% CI]	*p* value	ß [95% CI]	*p* value
Independents				
Constant	5.3		6.13	
Pre-pregnancy obesity	0.22 [0.017, 0.43]	**0.034**	0.041 [-0.11, 0.20]	0.60
GWG	0.022 [0.007, 0.037]	**0.004**	0.006 [-0.005, 0.018]	0.27
Primiparity	0.032 [-0.15, 0.22]	0.73	-0.29 [-0.43,- 0.15]	**<0.001**

^1^ Log-values

*Adjusted for maternal age, education, smoking, mode of delivery and newborn gestational age and sex.

**Adjusted for maternal age, education, smoking, mode of delivery and newborn gestational age, sex, and cord blood log C-peptide.

## Discussion

This is the first study to evaluate the association between cord blood C-peptide and IGF-I and newborn regional body composition assessed by DXA. Newborn fat-free mass was positively associated with C-peptide and IGF-I concentrations, and furthermore total fat mass and abdominal and arm and leg fat mass were positively associated with C-peptide. These associations were still present after adjustment for maternal factors such as age, education, smoking, parity, pre-pregnancy BMI and GWG. Our primary finding was, however, the divergent effect of IGF-I: arm and leg fat mass was positively associated with IGF-I, but there was no association between IGF-I and abdominal fat mass or total fat mass.

### Newborn regional body composition versus IGF-I and C-peptide

The finding of higher birth weights in association with higher cord blood levels of both C-peptide and IGF-I has previously been demonstrated [17,21]. IGF-I is important for normal fetal growth and may be a mediator related to development of childhood obesity and non-communicable diseases in adulthood [15,33–35]. The association between newborn regional body composition and IGF-I is unknown, and previous studies on the association between fetal fat mass and IGF-I are contradictive [36,37]. Another study in which body composition was assessed within 2 weeks of birth using DXA to assess newborn body composition found total fat mass to be related to IGF-I in 119 newborns [36]. In contrast, Beltrand et al. examined a cohort of neonates who were at risk of low birth weight and observed that newborn fat mass was closely associated with insulin levels, whereas no association was seen between fat mass and IGF-I. Despite data on regional body composition, there are no analyses on the association between regional fat distribution and cord blood insulin and IGF-I [37].

A priori, we hypothesized that the amount of abdominal fat mass would be associated with cord blood concentrations of IGF-I. This hypothesis was based on an vitro study of fat cells from 6-year-old children in whom the differentiation of visceral fat cells was more strongly associated with IGF-I concentration than the differentiation of subcutaneous fat cells [23] However, our findings point in the opposite direction, indicating that the effect of IGF-I on fat cells may be different in utero compared to childhood, and we propose that IGF-I stimulates peripheral fat tissue accumulation.

There are common denominators between third trimester intrauterine growth and growth between 0 and 6 months. Ong et al. report that infant linear growth (fat-free mass and length gain) is primarily associated with IGF-I and propose that infant adiposity is associated with insulin levels or other undetermined factors, and our observations support this view [38].

### C-peptide and IGF-I versus maternal factors

In our study, maternal obesity affected cord blood levels of C-peptide but not IGF-I, whereas cord blood C-peptide was related to GWG. Birth weight has previously been found to be associated with GWG [28] and with both maternal insulin sensitivity and the cord blood concentration of C-peptide [17]. Hence, the effect of GWG on cord blood C-peptide is likely to be mediated through maternal glucose. We have found no previous reports on the association between GWG and cord blood C-peptide.

We found clear differences in the body composition of the offspring of obese versus normal-weight mothers. A study has shown that maternal third trimester insulin-like growth factor-binding protein 1(IGFBP-1) was inversely associated with maternal pre-pregnancy BMI. Low concentrations of IGFBP-1 increase the bioavailability of maternal IGF-I, which increases placental nutrient transportation [39]. However, we suggest that newborn body composition is directly associated with pre-pregnancy obesity, and the effect is mediated not only through fetal IGF-I because we found no association between cord blood IGF-I and pre-pregnancy obesity. IGF-I concentration was lower in primiparas than in multiparas as reported by Vatten et al. The reason for this is not fully understood but may be related to a relative placental dysfunction in primiparas [21].

### Strengths and limitations

Due to failure to obtain cord blood samples, our cohort was limited to 174 cases, and this may induce a type II error. Our cohort was primarily recruited through the TOP study. The maternal TOP study intervention did not affect newborn body composition [32] nor did it affect cord blood concentration of C-peptide and IGF-I (data not shown). However, it is possible that the women who participated in this study had a general awareness regarding health issues that would not have been present if the cohort was randomly recruited [40]. It also may affect outcome that the normal weight women did not receive any intervention. Limitations to our study were that the obese mothers filled out the baseline questionnaires during the first trimester and normal-weight mothers during the third trimester and pre-pregnancy weight was self-reported in both obese and normal weight women.

Our cohort is rather homogenous, and this reduces the risk of bias. Our cohort consisted primarily of neonates with an increased risk of high birth weights. Fetal IGF-I is closely related to fetal weight, and therefore the relatively high mean birth weight in our cohort may be important when interpreting our results.

It is not possible to differentiate between subcutaneous and visceral fat mass with a DXA scan. It has been shown previously that DXA-estimated abdominal fat mass can be used as a proxy for visceral fat mass in cohort studies, although the method is less precise than magnetic resonance imaging [41,42]. This differentiation is important since it is the visceral fat mass that is linked to the development of the metabolic syndrome. Cooke et al. have used DXA to assess regional body composition in preterm and term born infants and determined intra-operator variability to approximately 1% [30]. There are no studies in newborns validating the use of DXA to assess regional body composition. We, however, used the same method as Cooke at al., and only included the scan if the paired weight between arm and legs agreed (±10%).

The concentration of cord blood hormones is affected by delivery. All participants in our study had deliveries without major newborn complications. Asphyxia is known to affect IGF-I, but no asphyctic newborns were included [43]. There were no statistical differences in mode of delivery between obese and normal weight women. All analyses were adjusted for mode of delivery.

## Conclusion

In this cohort of healthy newborns with obese and normal weight mothers both peripheral and central fat mass was associated with cord blood C-peptide. Fat-free mass and arm and leg fat mass were associated with cord blood IGF- I, whereas there was no association between the amount of abdominal fat mass and IGF-I. Hence, C-peptide may be a mediator of newborn adiposity whereas IGF-I levels are mainly related to linear growth. The long-term implications of these associations need to be explored in further studies.

## Supporting Information

S1 Protocol Supporting information: Original study protocol. (DOC)
